# Anger Expression in Negotiation: The Effects of Communication Channels and Anger Intensity

**DOI:** 10.3389/fpsyg.2022.879063

**Published:** 2022-06-02

**Authors:** Dongwon Yun, Heajung Jung

**Affiliations:** ^1^William F. Harrah College of Hospitality, University of Nevada, Las Vegas, Las Vegas, NV, United States; ^2^School of Business, Konkuk University, Seoul, South Korea

**Keywords:** anger expression, negotiation, communication channel, anger intensity, non-verbal cues

## Abstract

This research aimed to explore the effects of communication channels and anger intensity as factors determining how the expression of anger affects negotiation outcomes. Based on the “emotions as social information” (EASI) model and media richness theory, we tried to examine how anger expression influences both economic and psychological negotiation outcomes as a function of communication channels and explore its underlying mechanism. In Study 1, 470 participants were randomly assigned to one of the five experimental conditions-neutral, anger expression *via* text/emoticon/voice/video-and asked to participate in an online negotiation task. The results showed a significant main effect of communication channel; partner’s anger expression *via* communication channels richer in non-verbal cues (voice and video) led participants to make a higher concession and report lower satisfaction with negotiation and lower desire for future interaction with the same partner compared to anger expression *via* less rich channels (text and emoticon). The anger expression effects on psychological outcomes were partially explained by perceiver’s anger experience in response to anger display, which is consistent with the affective mechanism proposed by the EASI model. Study 2 examined whether the results of Study 1 could be attributable to the different levels of anger intensity perceived by the participants across different communication channels. Data analyses from 189 participants showed a significant main effect of anger intensity only with a desire for future interaction, but not with satisfaction and concession. The insignificant findings of the latter imply that the observed channel effect in Study 1 cannot be fully explained by the intensity effect.

## Introduction

Society has changed radically as advanced technologies have increasingly permeated our daily lives (ITU, 2017). One of the major changes has been the innovation in communication methods. The ways of human interaction have been reshaped by the proliferation and popularity of technology (Berger, 2013). With the invention of new telecommunication modes, such as smartphones, video calling, and voice over internet protocol (VoIP), it has become easier for people to communicate long distances without any physical limitations (Abeele et al., 2018).

The ongoing COVID-19 global pandemic has forced us to reject the traditional ways of communication to minimize health risks. In education, online classes and academic conferences using videoconferencing tools, such as Zoom, have become prevalent to enable learning without direct contact. In business, indirect communication has now become an essential part of organizational life and interpersonal interactions. Team meetings as well as training sessions for employees are being conducted online. These changes also affect negotiation as a form of official communication between stakeholders in and out of organizations. Negotiation refers to “a process of social interaction by which parties interdependently make decisions about how to distribute resources and/or resolve conflicts” (Thompson and Hastie, 1990). Given the interactive aspects of the negotiation, the search for the methods of effective communication might be a matter at hand. As online communication provides convenient interactions as an inevitable substitute for traditional communication channels, the impact of electronic media on negotiation behavior has begun to draw more attention from both researchers and practitioners (Galin et al., 2007; Geiger, 2020).

With research increasingly comparing the role of different communication channels, negotiation scholars have begun to explore the channel effect on the negotiation process and outcomes (Geiger and Parlamis, 2014). Most of this research has simply compared computer-mediated versus face-to-face communication (Giordano et al., 2007; Johnson et al., 2009; Chen and Tseng, 2016; Crossley et al., 2016). Online channels, despite their commonality as technology-aided communication tools, involve unique and differentiating features. One salient feature might be the amount of non-verbal cues that can be delivered through each channel. For example, instant messages are not as capable of delivering non-verbal cues—including facial expression, vocal tone, and body movements—as video calls in which individuals can see and hear each other. These differences are important since non-verbal cues are known to play an important role in delivering the message as well as its associated emotion (Rezabek and Cochenour, 1998; Walther and D’Addario, 2001; Pell et al., 2015). Given the importance of emotional exchange in negotiations [e.g., for strategic use of emotional display, see Tng and Au (2014)] and of the pivotal role of non-verbal cues in delivering emotions, categorizing communication channels in terms of their ability to deliver non-verbal cues and examining the role of anger display *via* different channels on negotiation outcomes will provide valuable insights and useful pointers for practitioners.

To bridge the existing gap in negotiation literature, we propose the research model examining how distinguished communication channels influence the effect of anger expression in negotiation. Specifically, this research explores the potential effects of online communication channels by comparing four computer-mediated communication methods for anger expression — text, emoticon, voice, and video. Scenario-based experimental designs are selected to test the causality in controlled settings. In Study 1, study participants are randomly assigned to one of the five communication channel conditions and asked to negotiate with their fictional partner. Both economic and psychological outcomes are measured and compared across experimental conditions. In the subsequent study, anger expression intensity is manipulated and examined in the context of video communication to rule out an alternative explanation of the communication channel effect. The study findings and theoretical and practical implications are discussed.

By exploring the proposed research model, we believe our study will contribute to the existing body of literature in several ways. First, it is still not clear whether anger expression *via* technology-aided channels would have the same effect as in traditional face-to-face interactions. Given that the strategic benefits of anger display during a negotiation have been reported (Van Kleef et al., 2004a,b; Sinaceur and Tiedens, 2006; Campagna et al., 2016; Gaspar et al., 2019), the anger effect should be re-examined under new contexts of communication. Furthermore, relatively few studies have examined whether there exists any significant difference among different online channels. The differences in the amount of non-verbal cues across communication channels are expected to yield different negotiation outcomes. The more non-verbal cues involved in emotional communication, the stronger the effect of emotional expression would become. In this research, we will break down technology-aided channels into four different categories based on their ability to deliver non-verbal cues and examine the effect of communication channels.

Second, this research outlines and examines the underlying mechanisms through which anger expression *via* different channels impacts negotiation outcomes. By means of incorporating media richness theory into EASI model, we attempt to explicate the phenomenon that cannot be fully explained by the current negotiation literature. We believe this research can make a significant and timely contribution to the field of negotiation research by applying communication theory and expanding our knowledge in the midst of a new era characterized with rapid technological advancement and unprecedented pandemic situation. In sum, the present research aims to explore whether anger expression would have differential effects on negotiation outcomes depending on online channels used for communication between negotiation partners.

## Theoretical Background and Hypothesis

### Anger Expression During Negotiation

The pervasiveness of negotiation in real life has promoted research exploring diverse factors that impact negotiation outcomes. One factor that has garnered significant attention from scholars is the emotions that are experienced and expressed in negotiation settings (Barry et al., 2004; Kopelman et al., 2006; Druckman and Olekalns, 2008; Jang and Bottom, 2022). Among diverse emotions, the expression of anger has been extensively studied in relation to the strategic use of emotions for achieving better deals (Kopelman et al., 2006; Tng and Au, 2014). Whether anger expression helps or hurts in negotiation can be a complex issue as negotiation performance can be measured either by economic (Pietroni et al., 2008; Adam and Brett, 2018) or psychological (Kopelman et al., 2006) outcomes. Prior research has reported that anger expression can elicit larger concessions from a negotiation partner leading to higher economic gain for the party who shows anger (Van Kleef et al., 2004a,b; Sinaceur and Tiedens, 2006; Jang and Bottom, 2022). When overall satisfaction with the negotiation process and outcome (Van Kleef et al., 2004a,b) and desire for future interaction with the same negotiation partner (Van Kleef and Côté, 2007; Pietroni et al., 2008; Van Kleef and De Dreu, 2010) were measured to understand psychological aspects of negotiation experience, anger display by a negotiation partner was reported to invoke negative emotional experiences, reducing the other party’s desire for future interaction and overall satisfaction with the negotiation process and outcome.

Notwithstanding an enhanced understanding of the role of anger display in negotiation settings, more nuanced aspects of anger expression have not yet been adequately investigated. We expect that exploring different communication channels employed for anger display can offer valuable insights into how anger expression could lead to different outcomes as a function of its carrier.

### Computer-Mediated Communication Channels

While face-to-face communication is the traditional, dominant manner of human interaction and is preferred for many reasons (Warschauer, 2013), it has some limitations, such as the necessity for the participants to be in the same place and time for synchronous communication (Chen and Tseng, 2016). Computer-mediated communication (CMC) is a viable alternative to face-to-face interaction since it transcends geographical and temporal limitations. CMC refers to all communications that use electronic devices as a medium and is a broader concept encompassing various types of technical tools, including text messages [Short Message Service(SMS)] *via* cell phone, VoIP (e.g., Skype or other voice call functions of messenger applications such as Telegram), and video calling such as Skype and Zoom (Kirwan, 2016).

Given the unique characteristics of each channel, CMC channels can be broadly grouped into three categories: text, auditory, and visual (refer to Geiger, 2020, Figure 3). As we are interested in emotional communication, in particular, we focus on communication tools that convey messages using (1) written texts, (2) spoken language along with auditory cues (e.g., vocal tone and volume), or (3) both verbal language and visual cues (e.g., facial expression and bodily movement), and can enable synchronous communications between two parties on par with traditional face-to-face interactions. Even though email is a widely used text-based channel, it is not included in our research due to its asynchronous nature.

First, SMS, which refers to a text messaging service of cell phone, smartphone, or internet applications (e.g., WhatsApp, Facebook Messenger, and Instagram direct message), might be one of the most familiar and frequently used channels due to its convenience. However, the text-based channels are limited, in that they cannot deliver auditory and visual non-verbal cues that face-to-face interactions can (Walther, 2015). Along with the common use of SMS, the use of emoticons is rapidly growing in personal as well as professional messages (Kaye et al., 2016). Despite its massive popularity and critical role in communicating one’s feelings, there are only a limited number of academic studies on its functions. The existing definitions of an emoticon emphasize the use of typographical symbols and the representation of one’s emotion (e.g., Rezabek and Cochenour, 1998; Huffaker and Calvert, 2005; Walther, 2015). The characteristics of an emoticon can play a complementary role of non-verbal cues when negotiators use text-based channels. In the absence of facial expressions and other visual cues, emoticons can substitute visual cues, or more specifically, facial expressions, in online communication despite their fictionality (Crystal, 2001; Walther and D’Addario, 2001; Zhou et al., 2017). Therefore, when using a text-based channel for emotional communication, emoticons can be utilized as a supplementary tool or in replacement of textual contents.

Given that texting and calling are the original ways of communication for cell phone users, they remain the most common modes (Laursen, 2012; Kemp, 2017). However, despite the use of the same device, the two communication methods differ significantly, as phone calls convey more non-verbal cues such as tone of voice, pitch, pauses, and breathing, all of which facilitate listening and understanding (Hopper, 1992). However, an auditory channel still lacks visual non-verbal cues that a video channel can offer. Video calls can be a viable alternative to face-to-face interaction since it does not require people to be in the same place simultaneously; yet it enables people to see each other and use their own voices. Video calling offers most of the benefits of face-to-face communication but is still not the same, as the non-verbal cues delivered through the video channel could suffer from reduced clarity, lack of vividness, or delay in message exchange due to technical issues.

Despite these differences among CMC channels, relatively fewer attempts have been made to differentiate these channels, especially in negotiation research (Sprecher, 2014; Schweinsberg et al., 2022). In the present study, communication channels for emotional expression are categorized into four different groups: *Text* refers to channels where emotional messages (“I’m angry”) are expressed *via* written text. *Emoticon* describes a communication method where emotion is displayed through images mimicking human facial expressions rather than typed words. *Voice* refers to channels in which emotion is expressed *via* spoken words carrying non-verbal cues related to vocal expressions (e.g., volume or tone). *Video* refers to video conferencing channels (e.g., Skype or Zoom), where emotions can be expressed and observed with visual cues such as a facial expression or bodily movement.

According to media richness theory (Daft and Lengel, 1986), these four CMC channels − Text, Emoticon, Voice, and Video − differ in their information richness, more specifically their capacity to deliver non-verbal cues. Non-verbal cues are trusted more than verbal messages in conflict situations because non-verbal behavior is considered unconscious (Ting-Toomey, 1999; Afifi, 2007) and seen as a reflection of less controlled and indeliberate processes by observers (Knapp et al., 1978). In the context of emotions, non-verbal cues were found to serve as a facilitator for emotional exchange and to strengthen the content of the message (Rezabek and Cochenour, 1998; Walther and D’Addario, 2001). Considering the importance of non-verbal cues in communications and the diversity of communication methods within the CMC category, it is noteworthy to compare CMC channels differing in non-verbal cue availability in a negotiation context.

**FIGURE 1:**
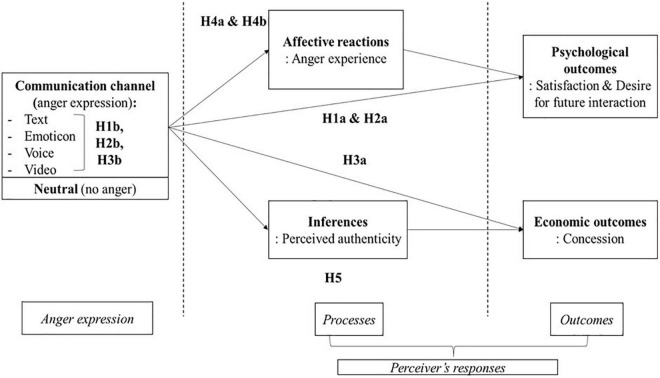
Research model and hypotheses.

As reviewed earlier, previous negotiation research has shown that anger display can result in positive economic outcomes such as more concession from negotiation partner in distributive negotiation and negative psychological/relational outcomes such as reduced satisfaction or future intention to negotiate with the same partner. In this research, we attempt to test these anger effects under different communication channels (see Figure 1). First, we expect to replicate the previous finding that anger expression negatively affects the psychological experiences of the perceiver compared to no anger display. Next, we would like to examine whether this negative relationship will be stronger when a richer communication channel is used for anger display. For example, compared to a negotiator who gets an angry text message, a negotiator who observes a partner’s anger expression through video call where anger is expressed through multiple cues including heightened voice, angry face, and aggressive gesture would report more negative relational outcomes, such as lower satisfaction about negotiation or lower intention for future interaction. Thus, we come to our first set of hypotheses:

*Hypothesis 1a: Participants in four anger expression conditions will report lower desire for future interaction with their partner who expressed anger compared to those in neutral condition.*
*Hypothesis 1b: There will be a significant difference in the level of future interaction desire depending on communication channel (text, emoticon, voice, and video) through which anger is expressed. Specifically, the more non-verbal cues included in the channel, the lower the desire for future interaction.*

*Hypothesis 2a: Participants in four anger expression conditions will report lower satisfaction with their negotiation with their partner who expressed anger compared to those in neutral condition.*
*Hypothesis 2b: There will be a significant difference in the level of satisfaction depending on communication channel (text, emoticon, voice, and video) through which anger is expressed. Specifically, the more non-verbal cues included in the channel, the lower the satisfaction.*

A well-established finding on the outcome of anger expression in negotiation is that people tend to make a larger concession to the person who is seen angry. Anger expression is usually perceived as a sign of aggression, incivility, and hostility, and unsuitable in most organizational contexts (Geddes and Callister, 2007). Yet during negotiations, observers perceive anger expression as a sign of toughness (Tiedens et al., 2000; Tiedens, 2001) and dissatisfaction with the offer, and tend to make more concessions to avoid an impasse (Van Kleef et al., 2004a; Pietroni et al., 2008). In line with existing studies, we expect that when negotiators perceive a partner’s anger expression, they will make a larger concession to reach an agreement. Additionally, the amount of concession will differ by communication channel such that the more non-verbal cues are employed for displaying anger, the stronger will be the anger effect on the amount of concessions. Our next set of hypotheses is as follows.

*Hypothesis 3a: Participants in four anger expression conditions will make larger concessions for their negotiation partner who expressed anger compared to those in neutral condition.*
*Hypothesis 3b: There will be a significant difference in the level of concession depending on communication channel (text, emoticon, voice, and video) through which anger is expressed. Specifically, the more non-verbal cues included in the channel, the larger the concession.*

### Emotions as Social Information Model

In order to understand the psychological mechanisms underlying the hypothesized channel effects, we need to trace back to prior research on functions and consequences of emotions from the perspective of interpersonal relationships (Frijda and Mesquita, 1994; Keltner and Haidt, 1999; Pietroni et al., 2009; Lerner et al., 2015; Olekalns and Druckman, 2015). First, it has been argued that emotional display tends to induce complementary or reciprocal emotional reactions from its observer, which are instrumental for responding to important social events (Keltner and Haidt, 1999). Second, the display of one’s emotion communicates valuable information to both oneself (Schwarz and Clore, 1983) and other people (Oatley and Johnson-Laird, 1987) by revealing one’s inner emotional state (Ekman, 1993). Lastly, emotions can serve as incentives or obstacles for others’ social behavior (Klinnert et al., 1983).

More recently, Emotions as Social Information (EASI) model proposed by Van Kleef (2009) is being widely applied to organizational and social phenomena including negotiation, leadership, and group decision making. It posits that emotions function as social information and influence counterparts *via* non-verbal (facial expression, body posture, or tone of voice) and/or verbal (language) channels, through an affective and inferential mechanism. The model formulates an individual’s overall decision-making process by utilizing emotions as an information source. It categorized the subsequent behaviors into two types: undesired and desired behaviors.

The affective mechanism describes the affective reactions of a perceiver who observes others’ emotional expressions. One’s emotional display can influence a perceiver’s attitude and behavior through several pathways, one of which concerns the affective reactions induced by emotional contagion, or the proclivity of catching others’ emotions (Hatfield et al., 1994). Emotional experiences *via* this process are referred to as reciprocal emotional experiences. For example, when an individual observes others’ non-verbal displays of emotions, such as facial, vocal, and postural expressions, those displays are mimicked; they produce congruent emotional states through afferent feedback processes (Hawk et al., 2012). These experiences through emotional contagion can influence one’s judgment and decision (Forgas, 1995). In addition to this is complementary emotional arousal (Keltner and Haidt, 1999), where complementary emotions are provoked when an individual observes certain emotions in others. Experiencing fear after observing another’s anger expression is an example of this complementary emotional experience (Dimberg and Öhman, 1996).

According to this affective reaction mechanism, when negotiators observe counterparts’ anger expression, they would experience reciprocal emotions by means of emotional contagion. The experience of anger as a response to counterpart’s anger expression falls into the affective reactions path of EASI model. Given the critical role of non-verbal displays during this contagion process, the extent to which the observer’s anger experience in response to others’ anger displays will increase with the richness of the communication channel employed. Specifically, when more non-verbal cues are provided signaling one’s anger, an observer will experience a greater level of anger than when less non-verbal cues are available, which will in turn negatively affect psychological outcomes in negotiation. This leads us to our next hypotheses on the mediating role of perceiver’s anger experience in the proposed channel effect.

*Hypothesis 4a: Participants’ anger experience will mediate the relationship between anger expression by different communication channels and desire for future interaction.*
*Hypothesis 4b: Participants’ anger experience will mediate the relationship between anger expression by different communication channels and satisfaction with negotiation.*

Emotions as Social Information model proposes that an inferential mechanism is activated when an individual tries to interpret the meaning from others’ affective expressions. Since people experience a particular emotion based on their appraisal of the current situation (Frijda et al., 1989; Lazarus, 1991; Scherer et al., 2001), observing a particular emotion in another person offers useful information about how they interpret the given situation (Van Kleef et al., 2011). An individual can engage in making a judgment on a counterpart’s emotional expression as a part of inference processes.

The distinct appraisal patterns of discrete emotions (Manstead et al., 1989; Smith et al., 1993) can provide resourceful information about the expresser (Van Kleef, 2009; Hareli and Hess, 2010). For instance, we can infer one’s inner state (Ekman, 1993), one’s orientation toward others (Hess et al., 2000), and appraisal of the situation (Manstead and Fischer, 2001) from displayed emotions. Thus, the negotiator’s evaluation of the authenticity of the counterpart’s emotional expression can be a process of information gathering for the inferences about the counterpart’s intentions based on the emotional expression. These inference processes can also affect the observer’s subsequent attitudes and behavior (Wang et al., 2012). When negotiators perceive their partner’s anger display as a genuine signal of inner state, they will infer that the partner is not satisfied with the situation and adjust their behavior accordingly such as offering larger concessions to their partner to avoid an impasse (Tng and Au, 2014). By contrast, when the negotiators infer that the anger display is inauthentic, they will assume that the counterparts are deliberately expressing anger as a way of bluffing to maximize benefits. In this context, the negotiator will feel unjustly treated (Allred et al., 1997) and take actions to prevent future injustice (Van Dijk et al., 2008). Thus, when lower-level authenticity is experienced, it will lead to less cooperative behavior such as smaller concessions.

*Hypothesis 5: Perceived authenticity of anger expression will mediate the relationship between anger expression by different communication channels and the amount of concession.*

## Research Design

### Research Overview

To address how the expression of anger *via* different communication channels affects both economic (e.g., concession: objective measurement of negotiation outcome in a distributive negotiation setting) and psychological (e.g., desire for future interaction and satisfaction with negotiation process) outcomes, this study employed a scenario method. Five different conditions in which anger expression by the negotiation partner is delivered through four different channels or anger is not explicitly expressed (i.e., neutral) were created and study participants were randomly assigned to one of these conditions. Participants’ responses to their negotiation partner’s anger display were collected and analyzed to test the channel effect. Study 2 is a post-hoc study to see if the differential effects of communication channels found in Study 1 could be due to differences in the level of perceived anger expression intensity, a possible alternative explanation. A low versus the intense level of anger expression during negotiation under the same video channel was manipulated in order to test the effect of anger expression intensity while holding the channel effect constant. All participants in Study 1 and Study 2 were recruited online and were compensated for their participation when the survey was successfully completed.

For statistical power, we used G*Power (Faul et al., 2009) analysis with medium effect size, *f*^2^ = 0.0625 at significance level α = 0.05 with a power of 0.80. The results recommended a sample size of 197 (Study 1) and 128 (Study 2) for each study. For both studies, more than the minimum sample size requirement was collected. It assures the test of possible three-way interaction effect by adding participants’ gender in the analysis (Dawson and Richter, 2006).

### Study 1: Communication Channel

#### Study Method

To test the hypotheses pertaining to the effects of communication channels on anger expression during negotiation, scenario-based negotiation tasks were presented to the participants. They read the same background information regarding the negotiation situation and received the same message from their partner *via* different communication channels. In Study 1, both economic and psychological outcomes of negotiation were assessed to investigate how anger expressed through different communication channels affected the negotiation outcomes.

#### Participants

In Study 1, online survey method was used to collect responses from participants. The link to the study was distributed through a survey agency to the public in South Korea. Participants were paid 1,000 Korean won (about US $1) as a reward for their participation and were informed that the respondents who successfully finished the survey would get a chance to join a lottery for extra reward. Of the 593 participants who passed the initial screening, 93 did not finish or withdrew from the survey, and 30 provided outlier responses, which left us with 470 participants for analysis. The average age of the participants was 37.07 years (SD = 9.65, *Min*_age_ = 20, *Max*_age_ = 59) and 60.2 percent of the sample was female (283 female, 187 male).

#### Procedure

To provide participants with familiar negotiation situations, we modified the negotiation task used by Schaerer et al. (2016). The original task describes a negotiation over a used coffee machine; we changed the item into a second-hand smartphone, a universal item of use and more frequently traded online than a coffee machine in South Korea. The study participants were instructed to play the role of seller and negotiate with a potential buyer online.

Once participants read a brief introduction to the study and signed a consent form, they first completed PANAS (Positive and Negative Affect Schedule) scale measuring their affective states. Next, they read the basic information about the negotiation situation and learned that the negotiation task consisted of three consecutive rounds and they (seller) had three chances to make an offer to their counterpart (buyer). They were also instructed to offer a fixed price (800,000 Korean won, which is about US $720) for the first two rounds to show toughness and, in response to the buyer’s anger expression, make a final offer of their choice in the final round. Before joining the negotiation session, participants were randomly assigned to one of the five study conditions (neutral, text, emoticon, voice, and video condition). Participants were led to believe that they interact with another participant playing the role of buyer through text messages, but in fact, they were exposed to pre-set messages including the amount of offer and anger expressing statement from the buyer.

To test the channel effect, the buyer’s anger was expressed *via* different channels depending on the assigned condition. In the *text* condition, a text message saying “I am angry about your offer” was sent to the participants, while in the *emoticon* condition, an angry-faced emoticon replaced the above message while the rest of the message was still delivered in a text message format. To create an anger message for the *voice* and *video* condition, we hired two confederates blind to the research hypotheses who were undergraduate students majoring in acting and trained them to read the message, “I’m angry about your offer” with non-verbal expressions. The confederates sighed and expressed their anger in a raised voice with additional facial expressions and body gestures, such as frowning and bending head. In the *voice* condition, only the audio of the recorded video was extracted and used for manipulation, whereas in the *video* condition, the whole video was shown to the study participants. To control for any potential gender effect, we intentionally hired one male and female confederates and randomly assign one of them to each participant. Table 1 summarizes how anger expression was manipulated for each channel.

**TABLE 1:** Five experimental conditions used in Study 1.

Condition		Manipulation method
No anger expression condition	Neutral	Participants get a text message from their partner that he/she cannot accept their offer.
Anger expression condition	Text	Participants get a text message from their partner that he/she cannot accept their offer and he/she feels angry about the offer.
	Emoticon	Participants get a text message from their partner that he/she cannot accept their offer, along with angry-face emoticon.
	Voice	Participants listen to a recorded message from their partner that he/she cannot accept their offer and he/she feels angry about the offer.
	Video	Participants watch a recorded video in which their partner says he/she cannot accept their offer and he/she feels angry about the offer.

After an angry message was delivered to the participants *via* different channels, their final responses to the message (i.e., final offer) were collected and analyzed. Lastly, participants answered questions intended to measure dependent variables such as satisfaction with the negotiation and desire for future interaction with the same partner, demographic information, and personality traits (i.e., agreeableness and extraversion).

#### Measures

##### Manipulation Check

To check whether our manipulation successfully delivered an angry message, our participants were asked to rate Van Kleef and De Dreu’s (2010) three items on a 9-point scale, from 1 (strongly disagree) to 9 (strongly agree). The items included statements such as: “During the negotiation, the buyer appeared to be angry/irritated/aggravated.” The three items showed internal consistency (Cronbach α = 0.95).

##### Desire for Future Interaction

To assess participants’ willingness for future negotiations with their counterparts, Van Kleef et al.’s (2004a) three items were used with a 7-point scale. The three items were: “I would be interested in negotiating again with this buyer,” “I would like to avoid future negotiation with the buyer,” and “I would like to do business with the same buyer in the future” (Cronbach α = 0.88).

##### Satisfaction With Negotiation

Two items from Van Kleef et al.’s (2004a) were used to measure the degree of satisfaction with negotiation after being translated into Korean using the back-translation method (Brislin, 1970). The items included “I am satisfied with the course of the negotiation,” and “I have a good feeling about the negotiation” (Cronbach α = 0.87) and participants responded using a 7-point scale.

##### Concession

The economic negotiation performance was measured by the amount of concessions made by the participants. As the respondents were instructed to offer a fixed price for the first two rounds, the concessions were calculated by subtracting the amount of the final offer from their first/second offer. The higher the amount of concessions made by participants, the less monetary gain obtained by them.

##### Anger Experience

The extent to which study participants experienced anger during the negotiation was measured by using Tng and Au’s (2014) three items on a 9-point scale, including “I felt anger/frustration/irritation toward the buyer during the negotiation,” (Cronbach α = 0.93).

##### Perceived Emotional Authenticity

The degree of perceived authenticity of anger expression by counterpart was measured by Tng and Au (2014) two items on a 7-point scale including “The emotion displayed by the buyer was sincere,” and “The emotion displayed by the negotiation counterpart is likely to be an honest reflection of what he or she was feeling” (Cronbach α = 0.84)^1^.

##### Control Variables

Based on the research by Kong (2015), both positive and negative affects were measured to control for participants’ general affective tendency; this is because the procedure of negotiation can be affected by the experience and expression of the participant’s positive (Kramer et al., 1993; Moore et al., 1999; Olekalns and Smith, 2009) and negative affect (Carnevale and Isen, 1986; Lerner et al., 2004). The validated PANAS scale translated into Korean by Lee et al. (2003) was used with a 5-point scale from 1 (not at all) to 5 (extremely). Among the 20 items of PANAS, nine assessed positive affect while the remaining measured negative affect. A prominent feature of this measure is that the term “alert” is categorized into negative affect, unlike in the original scale (Watson et al., 1988). With high reliability indices for positive (Cronbach α = 0.88) and negative affect (Cronbach α = 0.91) measures, the items were averaged for each measure and used in further analysis. In prior research, individual differences as a stable trait have been found to affect people’s cognition and behavior during negotiation.

As personality traits such as agreeableness and extraversion reflect one’s propensity to navigate the interpersonal aspects of one’s life (McCrae and Costa, 1987; Hofstee et al., 1992) and have been found to influence negotiation outcomes (Dimotakis et al., 2012; Wilson et al., 2016), they were included as control variables in this study. To measure these two personality traits, the translated version of the HEXACO-60 scale by Ashton and Lee (2009) was used in which 10 items measure each trait. The scales for agreeableness (Cronbach α = 0.79) and extraversion (Cronbach α = 0.88) showed acceptable reliability.

#### Results

##### Manipulation Check

One-way ANOVA analysis was conducted to check if there were significant differences in the participants’ perception of anger expression in their negotiation partner across the five experimental conditions. The omnibus ANOVA test revealed a significant difference by communication channel, *F*_(4, 465)_ = 113.92, *p* < 0.001. Planned contrast analyses revealed a significant difference between neutral and the other four conditions [*t*_(132.63)_ = −16.45, *p* < 0.001, *r*^2^ = 0.671] combined. Additionally, pairwise comparisons between neutral condition (*M* = 3.60, *SD* = 1.75) and each anger expression channel condition, yielded significant differences [Text: *M* = 5.97, *SD* = 1.71, *t*_(189.92)_ = −9.50, *r*^2^ = 0.322; Emoticon: *M* = 5.86, *SD* = 1.52, *t*_(179.84)_ = −9.33, *r*^2^ = 0.326; Voice: *M* = 7.79, *SD* = 1.20, *t*_(168.36)_ = −19.40, *r*^2^ = 0.691; Video: *M* = 7.56, *SD* = 1.48, *t*_(184.59)_ = −16.94, *r*^2^ = 0.609, *p* < 0.001]. However, there were no statistically significant differences between text and emoticon and between voice and video conditions [text vs. emoticon: *t*_(179.98)_ = 0.47, voice vs. video: *t*_(180.87)_ = 1.16].

To test the proposed hypotheses, a one-way ANCOVA analysis (channel: neutral, text, emoticon, voice, and video) was conducted where positive and negative affect, agreeableness, and extraversion were included as covariates. Furthermore, a series of planned contrasts analyses were performed to identify the differences between conditions. The coding scheme for contrast analysis is summarized in Table 2.

**TABLE 2:** Contrast analysis coding summary.

Coding for planned contrasts analysis		Condition				
		Neutral	Text	Emoticon	Voice	Video
A	Part of Helmert coding	1	−0.25	−0.25	−0.25	−0.25

B	Simple coding	−1	1	0	0	0
		−1	0	1	0	0
		−1	0	0	1	0
		−1	0	0	0	1

C	Part of repeated coding	0	1	−1	0	0
		0	0	1	−1	0
		0	0	0	1	−1
						

##### Desire for Future Interaction

As hypothesized, one-way ANCOVA analysis revealed a significant difference in one’s desire for future interaction with their negotiation partner by communication channel [*F*_(4, 465)_ = 4.68, *p* < 0.01, η^2^ = 0.39]. To test specific hypotheses, we used a part of Helmert coding (Keppel, 1991), which compared the neutral condition and the other four anger expression conditions combined. The contrast analysis result using Helmert coding (see row A in Table 2) showed that participants in the neutral condition reported a greater level of desire for future interaction with their partner compared to the four anger expression conditions, *t*_(465)_ = 3.46, *p* < 0.001, *r*^2^ = 0.025. To explore how each anger expression condition differs from the neutral condition, simple contrast coding was applied (see row B in Table 2). The analysis revealed that the level of desire for future interaction of respondents in the neutral condition significantly differed from the text [*t*_(465)_ = 2.22, *p* < 0.05, *r*^2^ = 0.010], voice [*t*_(465)_ = 3.68, *r*^2^ = 0.028] and video [*t*_(465)_ = 3.52, *r*^2^ = 0.026] conditions (*p* < 0.001), but not significantly from the emoticon condition (see Table 3 for mean scores by condition). Thus, Hypothesis 1a is partially supported.^2^

**TABLE 3:** Means/SDs by condition and one-way ANCOVA analysis results.

Channel	Sample size	Desire for future interaction	Satisfaction	Concession
		
		Mean (SD)	Mean (SD)	Mean (SD)
Neutral	96	3.53 (1.61)^a^	3.85 (1.45)^a^	9.57 (5.14)^a^
Text	96	2.98 (1.41)^bc^	3.41 (1.48)^ac^	9.97 (4.81)^a^
Emoticon	86	3.18 (1.31)^ab^	3.77 (1.31)^ab^	10.80 (5.77)^a^
Voice	97	2.76 (1.54)^c^	3.41 (1.62)^bc^	17.84 (11.04)^c^
Video	95	2.81 (1.53)^c^	3.34 (1.63)^c^	15.11 (11.40)^b^
*F*		4.68**	2.42*	17.90***
Partial η^2^		0.039	0.021	0.134

*p < 0.05, **p < 0.01, ***p < 0.001.

*The superscript letters next to SD indicate which values are significantly different from each other. Only the values with different letters are statistically different.*

Repeated coding was used (see C in Table 2) to test Hypothesis 1b, that the amount of non-verbal cues provided by the communication channel influences participants’ intention to interact with their partner in the future. A significant difference was only found between emoticon versus voice [*t*_(465)_ = 2.12, *r*^2^ = 0.010] and video [*t*_(465)_ = 2.04, *r*^2^ = 0.009] conditions (*p* < 0.05). Thus, Hypothesis 1b is also partially supported. In summary, while the participants in the neutral, text, and emoticon conditions reported a similar level of desire for future interaction, those in the voice and video conditions showed significantly lower desire levels than those in the former conditions.

##### Satisfaction With Negotiation

Satisfaction with the negotiation process and outcomes was analyzed as another psychological outcome variable. One-way ANCOVA analysis revealed a significant main effect of the communication channel [*F*_(4, 465)_ = 2.42, *p* < 0.05, η^2^ = 0.02]. Helmert coding (see row A in Table 2) for planned contrast analysis was used to test Hypothesis 2a. The analysis reports a significant difference in the level of satisfaction between those in neutral and in the four anger expression conditions combined, *t*_(465)_ = 2.05, *p* < 0.05, *r*^2^ = 0.009. Pairwise comparisons between neutral condition and each anger expression condition using simple coding (see row B in Table 2) show that participants in the neutral condition reported significantly higher satisfaction than those in the voice [*t*_(465)_ = −2.04, *p* < 0.05, *r*^2^ = 0.009] and video [*t*_(465)_ = −2.46, *p* < 0.05, *r*^2^ = 0.013] conditions, which indicates Hypothesis 2a is partially supported. Whether the level of satisfaction significantly changes when anger is delivered through a richer channel was tested by using repeated coding (see row C in Table 2). Contrary to our expectations, no significant difference between the pairs was found (*t*s < 1.77). In other words, the participants reported a similar level of satisfaction, regardless of the channel used for anger display. Thus, Hypothesis 2b is not supported.

##### Concession

The hypotheses on concession were tested *via* one-way ANCOVA analysis. Differences were found in the amount of concessions granted by a participant depending on the conditions assigned [*F*_(4, 465)_ = 17.90, *p* < 0.001, η^2^ = 0.13]. When Helmert coding (see row A in Table 2) was applied for planned contrasts analysis, consistent with Hypothesis 3a, the participants in the anger expression conditions conceded more than those in the neutral condition, *t*_(465)_ = −4.12, *p* < 0.001, *r*^2^ = 0.035. According to the contrast analysis using simple coding (see row B in Table 2), only the voice [*t*_(465)_ = 6.92, *r*^2^ = 0.093] and video [*t*_(465)_ = 4.64, *r*^2^ = 0.044] conditions differed in the degree of concessions from the neutral condition (*p* < 0.001). By applying repeated coding (see C in Table 2), the distinguished effects of channel on concession were explored. The results show that the participants in the neutral, text, and emoticon conditions made a similar amount of concessions to their counterparts (*t*s < 1.05) but made smaller concessions than those in the voice and video conditions. Additionally, the difference in the amount of concession between the voice and video conditions was significant [*t*_(465)_ = 2.23, *p* < 0.05, *r*^2^ = 0.011], although a significantly higher amount of concession was observed in the voice condition. In other words, an individual who observed anger *via* voice channel conceded more than those who interacted through video channel; thus, Hypothesis 3b is partially supported.

##### Mediation Analysis

To test the mediation hypotheses (4a, 4b, and 5), the bootstrapping method using model 4 in PROCESS Macro (Hayes, 2017) was performed by using seed number 61235. Consistent with the previous ANCOVA analysis, the same control variables were included. Given the coding strategy for categorical variables, we chose the control group as a reference group and dummy coded the variable for the analysis (see Table 4 for coding). The mediation effects of perceiver’s anger experience between anger expression by different channels and desire for future interaction were confirmed since zero are not included in the bootstrap confidence intervals (see Table 5). Thus, Hypothesis 4a is supported. The same method was applied to test hypothesis 4b. Consistent with the hypothesis, the indirect effects of anger experience linking anger expression by different channels with negotiator’s satisfaction were found to be significant (see Table 5). Thus, Hypothesis 4b is also supported.

**TABLE 4:** Coding of categorical variable (channel) for mediation analysis in Study 1.

Dummy coded variables	Condition				
	Neutral	Text	Emoticon	Voice	Video
X1	0	1	0	0	0
X2	0	0	1	0	0
X3	0	0	0	1	0
X4	0	0	0	0	1

*The neutral condition was used as a reference group.*

**TABLE 5:** Mediation effect of anger experience (Hypothesis 4a, 4b, and 5).

Outcome variable	Mediator		Effect	Boot SE	Boot LLCI	Boot ULCI
Desire for future interaction	Anger experience	X1	–0.5248	0.1299	–0.7848	–0.2752
		X2	–0.4115	0.1271	–0.6689	–0.1651
		X3	–0.8017	0.1468	–1.1025	–0.5211
		X4	–0.7428	0.1375	–1.0277	–0.4885

Satisfaction	Anger experience	X1	–0.4278	0.1126	–0.6563	–0.2152
		X2	–0.3355	0.1064	–0.5562	–0.1363
		X3	–0.6536	0.1275	–0.9253	–0.4142
		X4	–0.6056	0.1186	–0.8529	–0.3855

Concession	Perceived Authenticity	X1	–0.1182	0.2142	–0.5664	0.3114
		X2	0.3982	0.2284	0.0194	0.8965
		X3	0.3692	0.2532	–0.0778	0.9366
		X4	0.1957	0.2366	–0.2256	0.7030

When perceived authenticity of anger expression was tested as a mediator explaining the effect of channel on concession, the bootstrap CIs included zero, except the emoticon condition (see Table 5). For a better understanding of the results, we examined the effect of communication channels on perceived authenticity and the relationship between perceived authenticity and concession separately. The analysis showed that none of the four channel conditions are significantly related to perceived authenticity. However, perceived authenticity showed a positive relationship (β = 1.0295, SE = 0.2641) with concession [95% CI (0.5105, 1.5486)]. Even though the mediation effect was significant in the emoticon condition, it contradicts our prediction since it showed decreased authenticity than the control group. Thus, Hypothesis 5 is not supported.

#### Discussion

The results of Study 1 provide some support for the hypothesized effects of communication channels. Consistent with the literature (Van Kleef et al., 2004a; Van Kleef and Côté, 2007; Van Kleef and De Dreu, 2010), displayed anger resulted in favorable economic outcomes and unfavorable psychological outcomes, such as a lower desire for future interaction. Specifically, while anger expression in negotiation can make the counterpart concede more, it can also harm the relationship between the negotiators. The findings confirmed the notion that anger expression in negotiation is a double-edged sword. Furthermore, the EASI model was applied to understand how anger expression affects negotiation outcomes. Anger experience as a reaction to anger expression by a negotiation partner explained why anger expression through richer channels further weakens the level of satisfaction and the desire for future interaction.

Even though the hypotheses regarding the effects of communication channels were partially supported, some interesting findings were reported. First, a significant difference in desire for future interaction was observed between neutral and the four anger expression conditions combined. It was confirmed that observing a negotiation partner’s anger expression decreases the negotiator’s willingness for future engagement. However, when pairwise comparisons with the neutral condition were conducted, participants in the emoticon condition reported a similar level of desire for future interaction, indicating that expressing anger using emoticons is not seen as negative as it is through other channels. Furthermore, the finding that using angry emoticons does not yield negative psychological outcomes similar to anger expression through voice and video appears to negate the possibility that emoticons can be a replacement for non-verbal cues.

Second, the negative effect of anger expression on satisfaction with negotiation was successfully replicated. When the negotiation partner expressed anger explicitly, the observers reported lower satisfaction than those in the neutral condition. There was, however, no significant difference across the four different channel conditions in terms of satisfaction. In other words, when it comes to satisfaction, anger expression matters, regardless of the communication channel used.

Third, while a negotiator concedes more to the angry counterpart versus the neutral one, the amount of concession varied across communication channels. Participants in the neutral, text, and emoticon conditions made smaller concessions than those in the voice and video conditions. This demonstrates that anger expression richer in non-verbal cues —auditory and visual — is more effective in eliciting larger concessions from the other party compared to text-based anger expression. One unexpected but interesting finding is that the largest amount of concession was made in the voice channel. Considering that video utilizes visual cues as well as auditory cues, this result suggests the possibility that auditory cues may be the most critical element for augmenting the anger effect and adding visual cues may not make a noticeable additional contribution.

Fourth, the underlying mechanisms behind how anger expression *via* different channels affects negotiation outcomes were tested. Two mediating variables were proposed to explain the channel effects of anger expression on negotiation outcomes − economic and psychological. The indirect effects between communication channel and two psychological outcomes through perceiver’s anger experience in response to anger display of the negotiation partner were found to be significant, lending support to the affective reaction mechanism proposed by EASI model. However, perceived authenticity of anger expression (an inferential mechanism) did not receive support as a mechanism explaining the channel effect of anger expression on concessions. It suggests that other inferential mechanisms, such as inference of partner’s toughness, can be considered as an alternative explanation for the differential anger effect by communication channel.

While the observed effects of the communication channels are assumed to be related to the richness of non-verbal cues, participants could have perceived different levels of anger intensity across four different channels. In other words, anger expressed with more non-verbal cues could be perceived stronger in its intensity than anger expressed only *via* text or emoticon. Is it the intensity of anger expression that really matters? It is not clear yet whether the Study 1 findings can be simply explained by the intensity factor or the richness of non-verbal cues entails more than just the intensity. Since Study 1 is not self-sufficient to answer this question, further research is needed to tease the channel effect apart from the intensity effect.

### Study 2: Intensity of Anger Expression

Study 2 was designed to test a possible alternative explanation for Study 1 findings. The fact that richer channels conveying more non-verbal cues reinforced the anger effect on negotiation outcomes made us curious to know whether this finding could be possibly explained by the level of anger intensity perceived by participants. Besides an effort to provide an alternative explanation for the phenomenon, it also answers the call for future research exploring the anger intensity in negotiation (Hunsaker, 2017). To separate the intensity effect from the channel effect, Study 2 attempts to examine the role of anger expression intensity in isolation while controlling for the channel effect. A single communication channel with affluent non-verbal cues (i.e., video) was employed and the intensity of anger expression was manipulated by corresponding instructions to the actors, who played the role of negotiation partner in the video clip.

#### Participants

Study 2 was also based on an online survey, in which participants were randomly assigned to one of the four conditions created — intensity (high vs. low) × partner gender (male vs. female). At the onset of the study, participants read a brief description of the research and signed a consent form in which their right to withdraw from participation during the survey was explained. Respondents who successfully completed the survey were compensated 1,000 Korean won (about US $1) for their participation. Out of 223 people recruited for the survey, 23 respondents quit in the middle of the survey, and 11 provided outlier responses, leaving 189 participants for data analysis. The participants’ average age was 36.58 years (SD = 9.10, *Min*_age_ = 20, *Max*_age_ = 59) and 62.96 percent of them were female (119 female, 70 male). The participants who joined Study 1 were ruled out for additional participation for a pilot study and Study 2. Similarly, the participants in a pilot study were not qualified for Study 2 participation.

#### Procedure

Prior to Study 2, a pilot test was conducted to test the validity of intensity manipulation. Six conditions— 2 (negotiation partner gender: male vs. female) × 3 (intensity: high, moderate, and low) — were created and the same two confederates as in Study 1 were hired as actors for anger intensity manipulation. They were blind to the research purpose and hypotheses and were asked to express the same angry message with low, moderate, and high intensity. 57 participants joined the pilot test and were asked to rate the level of anger intensity depicted in the video clip. The average age of the participants was 31.39 (*SD* = 4.83, *Min*_age_ = 19, *Max*_age_ = 39), and 73.7 percent were female (42 female, 15 male). A one-way ANOVA analysis revealed that anger intensity was properly manipulated, *F*_(2,51)_ = 12.70, *p* < 0.001, η^2^ = 0.32. According to the results of planned contrast analyses, participants in the *high-intensity* condition (*M* = 7.20, *SD* = 1.06) perceived anger intensity significantly higher than those in the *low-intensity* condition [*M* = 5.61, *SD* = 1.46, *t*_(30.69)_ = 3.81, *p* < 0.001, *r*^2^ = 0.321]. Similarly, respondents in the *moderate-intensity* condition (*M* = 7.53, *SD* = 1.17) perceived a significantly higher level of anger intensity than those in the *low-intensity* condition [*M* = 5.61, *SD* = 1.46, *t*_(32.61)_ 4.38, *p* < 0.001, *r*^2^ = 0.370]. Since there was no significant difference between the high- and moderate-intensity conditions, low- and high-intensity videos were finally chosen for anger manipulation in Study 2.

In our main study, a modified version of Schaerer et al.’s (2016) negotiation task was used. To increase the generalizability of this research, we changed the individual buyer-seller exchange over a used cell phone into a business transaction between a store owner and a promotion agent. In the modified scenario, participants were asked to play the role of the store owner and negotiate with a service provider for flyer production and distribution as a part of new store promotion activities. The survey procedure of Study 2 was similar to that in Study 1.

#### Measures

The same measures used in Study 1 were reused in Study 2. The survey items were translated into Korean. The only difference is the manipulation check items as they directly asked how much anger intensity participants perceived in the interaction with their negotiation partner. Consistent with Study 1, the same control variables were included as covariates in the analysis.

#### Results

##### Manipulation Check

To check the effectiveness of intensity manipulation, participants’ reports of perceived intensity in their partner’s anger expression were compared between two intensity conditions. The *T*-test result showed a significant difference between the low-intensity condition (M = 5.95, SD = 1.50) and high-intensity condition [M = 7.21, SD = 1.57, *t*_(187)_ = −5.64, *p* < 0.001, *r*^2^ = 0.145], which proves successful manipulation of anger intensity.

##### Negotiation Outcomes

One-way ANCOVA analysis was conducted to investigate the main effect of the intensity of anger expression. The intensity effect was significant only with the desire for future interaction (see Table 6). For the two other outcome variables, concession and satisfaction with negotiation, there was no significant difference by anger intensity. In other words, regardless of the intensity level of anger display, participants reported similar levels of satisfaction and concession. To explore the difference in the willingness to interact with the same partner in the future between the low- and high-intensity conditions, a simple contrast analysis (contrast coding: -1, 1) was conducted. It was found that participants in the high-intensity condition (*M* = 3.35, *SD* = 1.54) showed a significantly lower level of desire for future interaction than those in low-intensity condition [*M* = 3.90, *SD* = 1.33, *t*_(189)_ = −2.65, *p* < 0.01, *r*^2^ = 0.036].

**TABLE 6:** One-way ANCOVA analysis summary in Study 2.

Anger Intensity	Sample size	Desire for future interaction	Satisfaction	Concession
		
		Mean (SD)	Mean (SD)	Mean (SD)
Low	97	3.90 (1.33)^a^	4.05 (1.29)^a^	20.91 (2.16)^a^
High	92	3.35 (1.54)^b^	3.75 (1.59)^a^	19.62 (2.86)^a^
*F*		6.96*	1.74	1.18
Partial η^2^		0.037	0.009	0.006

The superscript letters next to the mean scores indicate which values are significantly different from each other. Only the values with different letters are statistically different. *p < 0.01.

#### Discussion

Study 2 results provide somewhat complex answers to the question raised in Study 1. First, the intensity of anger expression had a significant effect on the desire for future interaction. This implies that in the case of long-term negotiations where negotiation deals are expected to be implemented over time — e.g., promotion activity (Study 2) vs. one-time purchase (Study 1) — and further negotiation opportunities and future interactions with the same partner are anticipated, the negotiator needs to be cautious not to show anger too intensely.

Interestingly, the amount of concession and satisfaction with negotiation did not change as a function of anger intensity. This indicates that the differential effect of anger expression by communication channel cannot be fully explained by perceived anger expression intensity. We can conclude that when displaying anger in negotiation, channel selection might be more critical than intensity control for economic outcomes or overall satisfaction with negotiation experience. If people care more about economic gains, showing anger using a channel rich in non-verbal cues might be more effective than showing anger using texts. In contrast, in long-term or multi-round negotiation situations where the desire for future interaction matters, it would be more desirable to keep anger expression at a lower intensity level and use text-based channels since it would be less harmful to relational outcomes.

## General Discussion and Conclusion

This research explores how the effect of anger expression on negotiation outcomes can be affected by the communication channel used for anger display. Drawing on media richness theory (Daft and Lengel, 1986), four channels – text, emoticon, voice, and video – were selected as representative media for emotional communication and the channel effects were examined in relation to both economic and psychological outcomes such as a desire for future interaction, negotiation satisfaction, and concession. Study 2 examined if the channel effect found in Study 1 could be explained by perceived anger intensity by solely manipulating anger expression intensity while holding the channel constant (i.e., video).

In Study 1, we successfully replicated prior findings that anger expression leads to more concession from a negotiation partner (Van Kleef et al., 2004a,b; Sinaceur and Tiedens, 2006; Wang et al., 2012; Jang and Bottom, 2022), lower desire for future negotiation with the same partner (Pietroni et al., 2008; Van Kleef and De Dreu, 2010), and reduced satisfaction with negotiation (Van Kleef et al., 2004a). Although anger expression using text/emoticon did not yield different negotiation outcomes from the neutral condition (except text condition with desire for future negotiation), when participants observed their negotiation partner’s anger through richer communication channels where more non-verbal cues are available (e.g., voice and video), the anger expression had stronger effects on all three negotiation outcomes. When four different channel conditions were compared to the neutral condition individually, these anger effects turned out to be more salient in the voice and video condition compared to the text or emoticon condition. While four communication channels did not show statistically significant stepwise changes in negotiation outcomes, the general pattern showing that richer communication channels reinforce the anger effects in negotiation settings is a fruitful observation we obtained in this research.

Moreover, the affective mechanism of the EASI model (Van Kleef, 2009) was empirically supported. The mediation analyses revealed that the channel effects of anger expression on a desire for future interaction and satisfaction could be explained by the negotiator’s anger experience in response to their partner’s anger display. However, the perceived authenticity of anger expression hypothesized to mediate the channel effect on concession, which corresponds to the inferential mechanism of the EASI model, turned out to be insignificant.

Study 2 results confirmed that Study 1 findings cannot be fully explained by the intensity effect. When anger was displayed *via* the same video channel and anger expression intensity was manipulated, no significant effect of anger intensity on satisfaction and concession was reported with the exception of desire for future interaction with the same partner.

In summary, the reported findings show that our research design as well as the psychological dynamics behind our observations are well-aligned with the EASI model. Our scholarly attempts confirmed that anger expression in negotiations yields favorable economic outcomes (Wang et al., 2012; Jang and Bottom, 2022) but accompanies relational harm as a return (Van Kleef et al., 2004a; Van Kleef and De Dreu, 2010). Notwithstanding some findings inconsistent with our postulated channel effects, we observed significant and consistent findings when comparing the text-based channels (text and emoticon) to the audio-based channels (voice and video). The results were not fully elucidated by media richness theory, but it still underpins the idea that more non-verbal cues aid the delivery of messages and emotions (Hopper, 1992). The rationale for the audio channel being the most effective in winning more concessions from negotiation partners still needs to be further investigated by exploring boundary conditions and applying other relevant theories.

### Theoretical and Practical Implications

This research contributes to the literature in several ways. First, in addition to confirming the prior findings regarding the effect of anger expression on negotiation, this study extends previous research by proving that the way anger is expressed − more specifically, channel selection for emotional communication − can bring out meaningfully different outcomes in negotiations. While previous studies on anger expression in negotiation did not systematically and fully examine the role of diverse communication channels, our research demonstrates that the effect of anger expression can vary by channel. The findings not only highlight the importance of communication channel selection for effective negotiation in the era of multiple computer-mediated communication methods available but also highlight the reason why we need to take communication methods into consideration in academic research for a more accurate understanding of the role of emotions in negotiation.

Second, this research attempted to systematically differentiate between CMC channels in the context of negotiation research. By focusing on the difference in non-verbal cue availability, four different channels in CMC were chosen: text, emoticon, voice, and video. As the channel shifted progressively from text to video, more non-verbal cues were displayed as suggested by media richness theory, and their potential effects on delivering anger expression were examined. Despite some insignificant effects, depending on the outcome variables, the results revealed that the effect of anger expression differs between text-based channels and non-verbal-based channels. The results confirmed that anger is more effectively delivered to the counterpart *via* richer channels where non-verbal cues, especially auditory cues, are readily available.

Third, the EASI model was empirically examined by testing two mechanisms (affective and inferential) using mediation analyses. The anger effects on two relational negotiation outcomes were significantly stronger when anger was displayed *via* richer channels (voice and video) and this channel effect proved to be mediated by the perceivers’ anger experience following anger display by their partner. In other words, participants who observed their counterpart’s anger expression through voice message or video experienced more intense anger as a reaction and this explained why they reported lower satisfaction and lower desire for future interaction. The channel effect of anger expression on concession as an economic outcome variable was expected to be related to the perceived authenticity of anger expression, which helps participants infer the true intention behind anger display. Yet, we found no evidence supporting the inferential mechanism in this research. Still, our attempt to combine media richness theory with the EASI model to hypothesize and empirically test the communication channel effects made an important step forward to a better understanding of the role of media.

Fourth, this research not only examined the communication channel effect but also tried to separate it from the intensity effect. In Study 1, we were not able to disregard the possibility that communication channels varying in their capacity to deliver non-verbal cues may impact the observer’s perception of anger intensity. To test the possibility that the difference between channels can be fully explained by the intensity effect, in Study 2, we tried to tease them apart by manipulating anger expression intensity in a single channel. We found that anger intensity mattered in the case of the desire for future interaction, but did not influence the other two outcome variables, satisfaction, and concession. This implies that the role of the communication channel in the anger effect cannot be simply attributable to the perceived intensity of anger.

The findings of this research provide some useful insights for practitioners in the field of negotiation. First, exploring the channel effect is a timely endeavor, considering that due to the pandemic, computer-mediated communication has become an inseparable part of our lives. This study would provide meaningful guidance for negotiators in choosing a communication channel. The same message (“I’m angry”) can be delivered *via* different channels using language/vocabulary (text), vocal expressions, and facial expressions/bodily postures. Given that different outcomes are expected depending on the communication channel employed, practitioners should be aware of the importance of channel selection when entering a negotiation.

Additionally, this research suggests that CMC channel selection should be done not only for convenience but also with careful consideration of negotiation goals and type (one-time vs. long-term). In other words, strategic selection of communication channels is crucial for attaining goals. Using the channel with affluent non-verbal cues such as video calls can be effective when the economic gain is the focus of the negotiation and negotiators would like to use anger to win more concessions from their partner. However, if the negotiators care about long-term relationships rather than short-term gains, anger displays through rich channels should be reconsidered because of the possibility of negative psychological consequences. Thus, strategic channel selection needs to be aligned with the purpose of the negotiation.

Lastly, negotiators should take into account that their decision-making process in negotiations is being influenced by affect (Cristofaro, 2020). In other words, field practitioners are required to understand the impact of making a hasty or emotional decision and regulate their emotional states when making a decision (Côté et al., 2013). Thus, negotiation skill training provided by an organization needs to cover emotional regulation, expression as well as recognition to cultivate a more emotionally versatile negotiator (Sharma et al., 2020). This caveat also aligns with the trend shifting from *bounded rationality* to *bounded emotionality* (Cristofaro, 2017) – the interactional relations between (un)conscious mental process and affective states are generating a new framework regarding rationality (Cristofaro, 2019).

### Limitations and Future Directions

This research is not without limitations. First, in spite of significant differences between text-based channels (text, emoticon) and non-verbal channels (voice, video), not all the hypothesized differences between channels were found to be significant. Specifically, there were no differences between text versus emoticon and voice versus video. Anger expression *via* text versus emoticon could be perceived similarly in that both channels rely on text message format. What was more surprising is that the participants did not show any difference in their perception of and reaction to anger between voice and video. In particular, contrary to our expectations, the amount of concession was greatest when anger was delivered through voice, not video. This is somewhat contradictory to our assumption that more non-verbal cues will make the anger display stronger. Would vocal expression of anger be sufficient for conveying an angry message and additional non-verbal cues like facial/bodily expression are just redundant? Or does it have something to do with our confederates’ lack of acting skills? If their facial expressions were perceived as less natural and inauthentic compared to their vocal expressions, anger display in the voice condition might have exerted more influence. Further research should be undertaken to compare and contrast the roles of vocal cues versus visual cues.

In addition, our study design where five different conditions were compared inevitably created a large number of hypotheses testing several group comparisons by contrast codings. This raises the issue of applying *p*-value correction before testing this multitude of hypotheses. Yet there is no firm consensus on using corrected *p*-values among statisticians. Some scholars insist that not performing adjustments would increase the possibility of type I error (e.g., Curran-Everett, 2000) while others argue that *p*-value corrections are not necessary (e.g., Rothman, 1990; Matsunaga, 2007; Althouse, 2016). Researchers defending the latter perspective touch on possible problems (Rubin, 2017a). One of the problems of conducting *p*-value correction is that it will increase the likelihood of type II error in return (Matsunaga, 2007). It accompanies a decrease in statistical power as well (Rothman, 1990). The opponents of the *p*-value corrections approach contend that type I error is dissipated to overall hypotheses rather than being localized to one or several hypotheses (Matsunaga, 2007; Rubin, 2017b). While we acknowledge both views are valid, we followed the no-adjustment approach as well as the other social science studies (Ding et al., 2020; Diachenko et al., 2021; Thériault et al., 2021) by conceding the potential problems of the taken approach.

Second, this research employed controlled experimental study designs to systematically compare the effect of different communication channels, which inevitably brings about the issue of ecological validity. Given the synchronous characteristics of the negotiation, inducing anger *via* pre-recorded voice and video may not be the most ideal method. Additionally, the techniques used in this study cannot be considered as reproducing full online interaction, in that only anger expression was presented *via* different channels while other contents were delivered *via* text message. Future research must develop more sophisticated experimental designs and methods incorporating the synchronous aspect of negotiation so that interactions with a negotiation partner proceed naturally while other factors are under control. Relatedly, the negotiation tasks given to the participants do not reflect the entire process of actual negotiation. The task was intentionally designed to reach an agreement while other prior research considered the possibility of impasses (Yip and Schweinsberg, 2017; Adam and Brett, 2018). Moreover, while most negotiation research used multi-issue tasks, the negotiation task used in this research dealt with a single issue, for the sake of simplicity. Considering these aspects, a more natural negotiation process should be devised in future research.

Third, the participant sample was recruited from South Korea, known as a hierarchical (Hofstede, 1980) and high-context (Ting-Toomey, 1985) culture where indirect communication styles (Morrison et al., 1994) are more common. The cultural characteristics of our sample might have affected the way of negotiating in this study and its results. Thus, to ensure the generalizability of our findings, further research needs to be conducted in different cultural settings such as western countries where using low-context (Hall, 1976) and egalitarian cultures (Hofstede, 1980) such as the United States.

Fourth, further attempts to explore the inferential mechanism of the EASI model are needed. We measured perceived authenticity of emotional expression as one potential mediator reflecting people’s inference about the true intention of anger expression, but the mediation analysis was not significant. Other variables, such as inferences regarding the counterpart’s reservation price or toughness, need to be considered to further investigate the inferential mechanism explaining the anger display effect in a negotiation setting.

Next, even though there were a few attempts to explore the effect of anger intensity in negotiation (Adam and Brett, 2018; Venkiteswaran and Sundarraj, 2020), they are relatively in a nascent stage despite the importance of the intensity factor in understanding the effect of anger expression more accurately. In this research, the confederates expressed anger in three different levels by changing their vocal tone/volume and facial expression, but participants in our pilot study failed to discern moderate-intensity anger from high-intensity anger. Since the video channel can deliver visual cues in addition to auditory cues, abundant non-verbal cues available could have made it difficult to clearly distinguish different intensity levels. For this reason, other communication channels can be easier for intensity manipulation. Considering the vocabularies used in expressing anger in the anger manipulation technique (Yun et al., 2020), future research might be able to use a different channel for successful intensity manipulation.

Sixth, while anger expression is the focus of this study, other emotions, such as guilt, anxiety, and happiness, are worth testing in the future as discrete emotions might elicit different outcomes in negotiation. It would be interesting if similar channel effects are observed when other emotions are expressed *via* different channels.

Lastly, in this research, only the steady-state emotion of participants was considered. An individual’s affective experience changes over time (Weiss and Cropanzano, 1996). Given the changing nature of emotional states, transitional emotions need to be included in future research. By exploring how an individual’s emotional experiences and expressions change over time during negotiation, more interesting findings can accrue.

## Data Availability Statement

The raw data supporting the conclusions of this article will be made available by the authors, without undue reservation.

## Ethics Statement

The studies involving human participants were reviewed and approved by the Konkuk University IRB. The patients/participants provided their written informed consent to participate in this study.

## Author Contributions

DY was responsible for contrivance of research idea, data collection and analysis, and literature review and writing. HJ developed the research idea and study design, took part in writing and editing, and advised the overall process of the research. Both authors contributed to the preparation of the manuscript and approved the final version of the manuscript.

## Conflict of Interest

The authors declare that the research was conducted in the absence of any commercial or financial relationships that could be construed as a potential conflict of interest.

## Publisher’s Note

All claims expressed in this article are solely those of the authors and do not necessarily represent those of their affiliated organizations, or those of the publisher, the editors and the reviewers. Any product that may be evaluated in this article, or claim that may be made by its manufacturer, is not guaranteed or endorsed by the publisher.

## Footnotes

1 Different scale points (e.g., 7-point, 9-point) were used for measures in this study, as we employed the original scales used in previously established studies. According to Leung (2011), no major difference in terms of internal structure such as means, SDs, Cronbach alphas, etc., was found between different scale points. In order to minimize any possible confusion in responding to questions, we grouped items using the same point Likert scale together when administering the survey.

2 The reason for some hypotheses being partially supported is that pairwise comparisons between the neutral condition and one of the anger expression conditions, or between two adjacent anger expression conditions, failed to reach a significant level.

## References

[B1] Abeele M. V. De Wolf R. Ling R. (2018). Mobile media and social space: how anytime, anyplace connectivity structures everyday life. *Media Commun.* 6 5–14. 10.17645/mac.v6i2.1399

[B2] Adam H. Brett J. M. (2018). Everything in moderation: the social effects of anger depend on its perceived intensity. *J. Exp. Soc. Psychol.* 76 12–18. 10.1016/j.jesp.2017.11.014

[B3] Afifi W. A. (2007). *Nonverbal Communication.* Mahwah, NJ: Lawrence Erlbaum Associates Publishers.

[B4] Allred K. G. Mallozzi J. S. Matsui F. Raia C. P. (1997). The influence of anger and compassion on negotiation performance. *Organ. Behav. Hum. Decis. Process.* 70 175–187. 10.1006/obhd.1997.2705

[B5] Althouse A. D. (2016). Adjust for multiple comparisons? It’s not that simple. *Ann. Thorac. Surg.* 101 1644–1645. 10.1016/j.athoracsur.2015.11.024 27106412

[B6] Ashton M. C. Lee K. (2009). The HEXACO–60: a short measure of the major dimensions of personality. *J. Pers. Assess.* 91 340–345. 10.1080/00223890902935878 20017063

[B7] Barry B. Fulmer I. S. Van Kleef G. (2004). “I laughed, I cried, I settled: the role of emotion in negotiation,” in *The Handbook of Negotiation and Culture: Theoretical Advances and Cross–Cultural Perspectives*, eds Gelfand M. J. Brett J. M. (Palo Alto, CA: Stanford University Press), 71–94.

[B8] Berger J. (2013). Beyond viral: interpersonal communication in the internet age. *Psychol. Inq.* 24 293–296. 10.1080/1047840x.2013.842203

[B9] Brislin R. W. (1970). Back-translation for cross-cultural research. *J. Cross Cult. Psychol.* 1 185–216. 10.1037/a0021453 21038953

[B10] Campagna R. L. Mislin A. A. Kong D. T. Bottom W. P. (2016). Strategic consequences of emotional misrepresentation in negotiation: the blowback effect. *J. Appl. Psychol.* 101:605. 10.1037/apl0000072 26653531

[B11] Carnevale P. J. Isen A. M. (1986). The influence of positive affect and visual access on the discovery of integrative solutions in bilateral negotiation. *Organ. Behav. Hum. Decis. Process.* 37 1–13. 10.1016/0749-5978(86)90041-5

[B12] Chen I. S. Tseng F. T. (2016). The relevance of communication media in conflict contexts and their effectiveness: a negotiation experiment. *Comput. Hum. Behav.* 59 134–141. 10.1016/j.chb.2016.01.039

[B13] Côté S. Hideg I. Van Kleef G. A. (2013). The consequences of faking anger in negotiations. *J. Exp. Soc. Psychol.* 49 453–463. 10.1016/j.jesp.2012.12.015

[B14] Cristofaro M. (2017). Herbert Simon’s bounded rationality: its historical evolution in management and cross-fertilizing contribution. *J. Manag. History* 23 170–190. 10.1108/jmh-11-2016-0060

[B15] Cristofaro M. (2019). The role of affect in management decisions: a systematic review. *Eur. Manag. J.* 37 6–17. 10.1016/j.emj.2018.12.002

[B16] Cristofaro M. (2020). “I feel and think, therefore I am”: an affect-cognitive theory of management decisions. *Eur. Manag. J.* 38 344–355. 10.1016/j.emj.2019.09.003

[B17] Crossley L. Woodworth M. Black P. J. Hare R. (2016). The dark side of negotiation: examining the outcomes of face-to-face and computer-mediated negotiations among dark personalities. *Pers. Individ. Differ.* 91 47–51. 10.1016/j.paid.2015.11.052

[B18] Crystal D. (2001). *Language and the Internet.* Cambridge: Cambridge University Press.

[B19] Curran-Everett D. (2000). Multiple comparisons: philosophies and illustrations. *Am. J. Physiol. Regul. Integr. Comp. Physiol.* 279, R1–R8. 10.1152/ajpregu.2000.279.1.R1 10896857

[B20] Daft R. L. Lengel R. H. (1986). Organization information requirements, media richness, and structural design. *Manag. Sci.* 32 554–571. 10.1287/mnsc.32.5.554 19642375

[B21] Dawson J. F. Richter A. W. (2006). Probing three-way interactions in moderated multiple regression: development and application of a slope difference test. *J. Appl. Psychol.* 91:917. 10.1037/0021-9010.91.4.917 16834514

[B22] Diachenko M. Smith K. K. Fjorback L. Hansen N. V. Linkenkaer-Hansen K. Pallesen K. J. (2021). Pre-retirement employees experience lasting improvements in resilience and well-being after mindfulness-based stress reduction. *Front. Psychol.* 12:699088. 10.3389/fpsyg.2021.699088 34335417 PMC8321239

[B23] Dimberg U. Öhman A. (1996). Behold the wrath: psychophysiological responses to facial stimuli. *Motiv. Emot.* 20 149–182. 10.1007/bf02253869

[B24] Dimotakis N. Conlon D. E. Ilies R. (2012). The mind and heart (literally) of the negotiator: personality and contextual determinants of experiential reactions and economic outcomes in negotiation. *J. Appl. Psychol.* 97 183–193. 10.1037/a0025706 21967294

[B25] Ding Y. Li D. Li X. Xiao J. Zhang H. Wang Y. (2020). Profiles of adolescent traditional and cyber bullying and victimization: the role of demographic, individual, family, school, and peer factors. *Comput. Hum. Behav.* 111:106439. 10.1016/j.chb.2020.106439

[B26] Druckman D. Olekalns M. (2008). Emotions in negotiation. *Group Decis. Negot.* 17 1–11. 10.1007/s10726-007-9091-9 PMC7380450 32764846

[B27] Ekman P. (1993). Facial expression and emotion. *Am. Psychol.* 48 384–392. 8512154 10.1037//0003-066x.48.4.384

[B28] Faul F. Erdfelder E. Buchner A. Lang A. G. (2009). Statistical power analyses using G* Power 3.1: tests for correlation and regression analyses. *Behav. Res. Methods* 41 1149–1160. 10.3758/BRM.41.4.1149 19897823

[B29] Forgas J. P. (1995). Mood and judgment: the affect infusion model (AIM). *Psychol. Bull.* 117 39–66. 10.1037/0033-2909.117.1.39 7870863

[B30] Frijda N. H. Mesquita B. (1994). “The social roles and functions of emotions,” in *Emotion and Culture: Empirical Studies of Mutual Influenced*, eds Kitayama S. Marcus H. (Washington, DC: American Psychological Association), 51–87. 10.1037/10152-002

[B31] Frijda N. H. Kuipers P. Ter Schure E. (1989). Relations among emotion, appraisal, and emotional action readiness. *J. Pers. Soc. Psychol.* 57 212–228. 10.1037/0022-3514.57.2.212

[B32] Galin A. Gross M. Gosalker G. (2007). E-negotiation versus face-to-face negotiation what has changed–if anything? *Comput. Hum. Behav.* 23 787–797. 10.1016/j.chb.2004.11.009

[B33] Gaspar J. P. Methasani R. Schweitzer M. (2019). Fifty shades of deception: characteristics and consequences of lying in negotiations. *Acad. Manag. Perspect.* 33 62–81. 10.5465/amp.2017.0047

[B34] Geddes D. Callister R. R. (2007). Crossing the line (s): a dual threshold model of anger in organizations. *Acad. Manag. Rev.* 32 721–746. 10.5465/amr.2007.25275495

[B35] Geiger I. (2020). From letter to twitter: a systematic review of communication media in negotiation. *Group Decis. Negot.* 29 207–250. 10.1007/s10726-020-09662-6

[B36] Geiger I. Parlamis J. (2014). Is there more to email negotiation than email? The role of email affinity. *Comput. Hum. Behav.* 32 67–78. 10.1016/j.chb.2013.11.016

[B37] Giordano G. A. Stoner J. S. Brouer R. L. George J. F. (2007). The influences of deception and computer-mediation on dyadic negotiations. *J. Comput. Mediat. Commun.* 12 362–383. 10.1111/j.1083-6101.2007.00329.x

[B38] Hall E. T. (1976). *Beyond Culture.* New York, NY: Anchor Press.

[B39] Hareli S. Hess U. (2010). What emotional reactions can tell us about the nature of others: an appraisal perspective on person perception. *Cogn. Emot.* 24 128–140. 10.1080/02699930802613828

[B40] Hatfield E. Cacioppo R. T. Rapson R. L. (1994). *Emotional Contagion.* New York, NY: Cambridge University Press.

[B41] Hawk S. T. Fischer A. H. Van Kleef G. A. (2012). Face the noise: embodied responses to nonverbal vocalizations of discrete emotions. *J. Pers. Soc. Psychol.* 102:796. 10.1037/a0026234 22059840

[B42] Hayes A. F. (2017). *Introduction to Mediation, Moderation, and Conditional Process Analysis: A Regression-Based Approach.* New York, NY: Guilford Publications.

[B43] Hess U. Blairy S. Kleck R. E. (2000). The influence of facial emotion displays, gender, and ethnicity on judgments of dominance and affiliation. *J. Nonverb. Behav.* 24 265–283.

[B44] Hofstede G. (1980). Culture and organizations. *Int. Stud. Manag. Organ.* 10 15–41.

[B45] Hofstee W. K. De Raad B. Goldberg L. R. (1992). Integration of the big five and circumplex approaches to trait structure. *J. Pers. Soc. Psychol.* 63:146. 10.1037//0022-3514.63.1.146 1494982

[B46] Hopper R. (1992). *Telephone Conversation*, Vol. 724. Bloomington, IN: Indiana University Press.

[B47] Huffaker D. A. Calvert S. L. (2005). Gender, identity, and language use in teenage blogs. *J. Comput. Mediat. Commun.* 10:JCMC10211.

[B48] Hunsaker D. A. (2017). Anger in negotiations: a review of causes, effects, and unanswered questions. *Negot. Conflict Manag. Res.* 10 220–241. 10.1111/ncmr.12096

[B49] ITU (2017). *ICT Facts and Figures 2017.* Available online at: https://www.itu.int/en/ITU-D/Statistics/Documents/facts/ICTFactsFigures2017.pdf (accessed March 30, 2017).

[B50] Jang D. Bottom W. P. (2022). Tactical anger in negotiation: the expresser’s perspective. *J. Behav. Decis. Mak.* 35:e2246.

[B51] Johnson N. A. Cooper R. B. Chin W. W. (2009). Anger and flaming in computer-mediated negotiation among strangers. *Decis. Support Syst.* 46 660–672. 10.1016/j.dss.2008.10.008

[B52] Kaye L. K. Wall H. J. Malone S. A. (2016). “Turn that frown upside-down”: a contextual account of emoticon usage on different virtual platforms. *Comput. Hum. Behav.* 60 463–467. 10.1016/j.chb.2016.02.088

[B53] Keltner D. Haidt J. (1999). Social functions of emotions at four levels of analysis. *Cogn. Emot.* 13 505–521. 10.1080/026999399379168

[B54] Kemp S. (2017). *Digital in 2017: Global Overview. Technical Report.* Available online at: https://wearesocial.com/blog/2017/01/digital-in-2017-global-overview (accessed March 30, 2017).

[B55] Keppel G. (1991). *Design and Analysis: A Research Handbook*, 3rd Edn. Upper Saddle River, NJ: Prentice-Hall.

[B56] Kirwan G. (2016). “Computer mediated communication and online media,” in *An Introduction to Cyberpsychology*, eds Connolly I. Palmer M. Barton H. Kirwan G. (London: Routledge).

[B57] Klinnert M. D. Campos J. J. Sorce J. F. Emde R. N. Svejda M. (1983). “Emotions as behavior regulators: social referencing in infancy,” in *Emotions in Early Development*, eds Plutchik R. Kellerman H. (New York, NY: Academic Press), 57–86. 10.1016/b978-0-12-558702-0.50009-1

[B58] Knapp M. L. Wiemann J. M. Daly J. A. (1978). Nonverbal communication: issues and appraisal. *Hum. Commun. Res.* 4 271–280. 10.1111/j.1468-2958.1978.tb00616.x

[B59] Kong D. T. (2015). Narcissists’ negative perception of their counterpart’s competence and benevolence and their own reduced trust in a negotiation context. *Pers. Individ. Differ.* 74 196–201. 10.1016/j.paid.2014.10.015

[B60] Kopelman S. Rosette A. S. Thompson L. (2006). The three faces of Eve: strategic displays of positive, negative, and neutral emotions in negotiations. *Organ. Behav. Hum. Decis. Process.* 99 81–101. 10.1016/j.obhdp.2005.08.003

[B61] Kramer R. M. Newton E. Pommerenke P. L. (1993). Self-enhancement biases and negotiator judgment: effects of self-esteem and mood. *Organ. Behav. Hum. Decis. Process.* 56 110–133. 10.1006/obhd.1993.1047

[B62] Laursen D. (2012). Sequential organization of text messages and mobile phone calls in interconnected communication sequences. *Discourse Commun.* 6 83–99. 10.1177/1750481311432517

[B63] Lazarus R. S. (1991). Cognition and motivation in emotion. *Am. Psychol.* 46 352–367. 2048794 10.1037//0003-066x.46.4.352

[B64] Lee H. Kim E. Lee M. (2003). A validation study of Korea positive and negative affect schedule: the PANAS scales. *Korean J. Clin. Psychol.* 22 935–946.

[B65] Lerner J. S. Li Y. Valdesolo P. Kassam K. S. (2015). Emotion and decision making. *Annu. Rev. Psychol.* 66 799–823. 25251484 10.1146/annurev-psych-010213-115043

[B66] Lerner J. S. Small D. A. Loewenstein G. (2004). Heart strings and purse strings: carryover effects of emotions on economic decisions. *Psychol. Sci.* 15 337–341. 10.1111/j.0956-7976.2004.00679.x 15102144

[B67] Leung S. O. (2011). A comparison of psychometric properties and normality in 4-, 5-, 6-, and 11-point Likert scales. *J. Soc. Serv. Res.* 37 412–421.

[B68] Manstead A. S. R. Fischer A. H. (2001). “Social appraisal: the social world as object of and Influence on appraisal processes,” in *Appraisal Processes in Emotion: Theory, Research, Application*, eds Scherer K. R. Schorr A. Johnstone T. (New York, NY: Oxford University Press), 221–232.

[B69] Manstead A. S. R. Tetlock P. E. Manstead T. (1989). Cognitive appraisals and emotional experience: further evidence. *Cogn. Emot.* 3 225–239. 10.1080/02699938908415243

[B70] Matsunaga M. (2007). Familywise error in multiple comparisons: disentangling a knot through a critique of O’Keefe’s arguments against alpha adjustment. *Commun. Methods Meas.* 1 243–265. 10.1080/19312450701641409

[B71] McCrae R. R. Costa P. T. (1987). Validation of the five-factor model of personality across instruments and observers. *J. Pers. Soc. Psychol.* 52, 81–90. 10.1037//0022-3514.52.1.81 3820081

[B72] Moore D. A. Kurtzberg T. R. Thompson L. L. Morris M. W. (1999). Long and routes to success in electronically mediated negotiations: group affiliations and good vibrations. *Organ. Behav. Hum. Decis. Process.* 77 22–43. 10.1006/obhd.1998.2814 9924140

[B73] Morrison T. Conaway W. A. Borden G. A. Koehler H. (1994). *Kiss, Bow, or Shake Hands: How to do Business in Sixty Countries.* Holbrook, MA: Adams Media Corporation, 456.

[B74] Oatley K. Johnson-Laird P. N. (1987). Towards a cognitive theory of emotions. *Cogn. Emot.* 1 29–50. 10.1080/02699938708408362

[B75] Olekalns M. Druckman D. (2015). “With feeling: how emotions shape negotiation,” in *Emotion in Group Decision and Negotiation*, ed. Martinovsky B. (Dordrecht: Springer), 33–50. 10.1007/978-94-017-9963-8_2

[B76] Olekalns M. Smith P. L. (2009). Mutually dependent: power, trust, affect and the use of deception in negotiation. *J. Bus. Ethics* 85 347–365. 10.1007/s10551-008-9774-4

[B77] Pell M. D. Rothermich K. Liu P. Paulmann S. Sethi S. Rigoulot S. (2015). Preferential decoding of emotion from human non-linguistic vocalizations versus speech prosody. *Biol. Psychol.* 111 14–25. 10.1016/j.biopsycho.2015.08.008 26307467

[B78] Pietroni D. Van Kleef G. A. De Dreu C. K. Pagliaro S. (2008). Emotions as strategic information: effects of other’s emotional expressions on fixed-pie perception, demands, and integrative behavior in negotiation. *J. Exp. Soc. Psychol.* 44 1444–1454. 10.1016/j.jesp.2008.06.007

[B79] Pietroni D. Van Kleef G. A. Rubaltelli E. Rumiati R. (2009). When happiness pays in negotiation. *Mind Soc.* 8 77–92.

[B80] Rezabek L. Cochenour J. (1998). Visual cues in computer-mediated communication: supplementing text with emoticons. *J. Visual Liter.* 18 201–215. 10.1080/23796529.1998.11674539

[B81] Rothman K. J. (1990). No adjustments are needed for multiple comparisons. *Epidemiology* 43–46. 2081237

[B82] Rubin M. (2017a). An evaluation of four solutions to the forking paths problem: adjusted alpha, preregistration, sensitivity analyses, and abandoning the Neyman-Pearson approach. *Rev. Gen. Psychol.* 21 321–329. 10.1037/gpr0000135

[B83] Rubin M. (2017b). Do p values lose their meaning in exploratory analyses? It depends how you define the familywise error rate. *Rev. Gen. Psychol.* 21 269–275. 10.1037/gpr0000123

[B84] Schaerer M. Loschelder D. D. Swaab R. I. (2016). Bargaining zone distortion in negotiations: the elusive power of multiple alternatives. *Organ. Behav. Hum. Decis. Process.* 137 156–171. 10.1016/j.obhdp.2016.09.001

[B85] Scherer K. R. Schorr A. Johnstone T. (eds.) (2001). *Appraisal Processes in Emotion: Theory, Methods, Research*, Vol. 14. New York, NY: Oxford University Press.

[B86] Schwarz N. Clore G. L. (1983). Mood, misattribution, and judgments of well-being: informative and directive functions of affective states. *J. Pers. Soc. Psychol.* 45 513–523. 10.1080/02699931.2021.2023108 34978262

[B87] Schweinsberg M. Thau S. Pillutla M. M. (2022). Negotiation impasses: types, causes, and resolutions. *J. Manag.* 48 49–76. 10.1177/01492063211021657

[B88] Sharma S. Elfenbein H. A. Sinha R. Bottom W. P. (2020). The effects of emotional expressions in negotiation: a meta-analysis and future directions for research. *Hum. Perform.* 33 331–353. 10.1080/08959285.2020.1783667

[B89] Sinaceur M. Tiedens L. Z. (2006). Get mad and get more than even: when and why anger expression is effective in negotiations. *J. Exp. Soc. Psychol.* 42 314–322. 10.1016/j.jesp.2005.05.002

[B90] Smith C. A. Haynes K. N. Lazarus R. S. Pope L. K. (1993). In search of the “hot” cognitions: attributions, appraisals, and their relation to emotion. *J. Pers. Soc. Psychol.* 65 916–929. 10.1037//0022-3514.65.5.916 8246115

[B91] Sprecher S. (2014). Initial interactions online-text, online-audio, online-video, or face-to-face: effects of modality on liking, closeness, and other interpersonal outcomes. *Comput. Hum. Behav.* 31 190–197. 10.1016/j.chb.2013.10.029

[B92] Thériault R. Olson J. A. Krol S. A. Raz A. (2021). Body swapping with a Black person boosts empathy: using virtual reality to embody another. *Q. J. Exp. Psychol.* 74, 2057–2074. 10.1177/17470218211024826 34049469 PMC8532211

[B93] Thompson L. Hastie R. (1990). Social perception in negotiation. *Organ. Behav. Hum. Decis. Process.* 47 98–123. 10.1016/0749-5978(90)90048-e

[B94] Tiedens L. Z. (2001). The effect of anger on the hostile inferences of aggressive and nonaggressive people: specific emotions, cognitive processing, and chronic accessibility. *Motiv. Emot.* 25 233–251.

[B95] Tiedens L. Z. Ellsworth P. C. Mesquita B. (2000). Sentimental stereotypes: emotional expectations for high-and low-status group members. *Pers. Soc. Psychol. Bull.* 26 560–575. 10.1177/0146167200267004

[B96] Ting-Toomey S. (1985). “Toward a theory of conflict and culture,” in *Communication, Culture and Organizational Processes*, eds Gudykunst W. Stewart L. Ting-Toomey S. (Beverly Hills, CA: Sage).

[B97] Ting-Toomey S. (1999). *Communicating Across CULTURES.* New York, NY: Guilford.

[B98] Tng H. Y. Au A. K. (2014). Strategic display of anger and happiness in negotiation: the moderating role of perceived authenticity. *Negot. J.* 30 301–327. 10.1111/nejo.12062

[B99] Van Dijk E. Van Kleef G. A. Steinel W. Van Beest I. (2008). A social functional approach to emotions in bargaining: when communicating anger pays and when it backfires. *J. Pers. Soc. Psychol.* 94 600–614. 10.1037/0022-3514.94.4.600 18361674

[B100] Van Kleef G. A. (2009). How emotions regulate social life: the emotions as social information (EASI) model. *Curr. Direct. Psychol. Sci.* 18 184–188. 10.1111/j.1467-8721.2009.01633.x

[B101] Van Kleef G. A. Côté S. (2007). Expressing anger in conflict: when it helps and when it hurts. *J. Appl. Psychol.* 92:1557. 18020796 10.1037/0021-9010.92.6.1557

[B102] Van Kleef G. A. De Dreu C. K. (2010). Longer-term consequences of anger expression in negotiation: retaliation or spillover? *J. Exp. Soc. Psychol.* 46 753–760.

[B103] Van Kleef G. A. De Dreu C. K. Manstead A. S. (2004a). The interpersonal effects of anger and happiness in negotiations. *J. Pers. Soc. Psychol.* 86 57–76. 14717628 10.1037/0022-3514.86.1.57

[B104] Van Kleef G. A. De Dreu C. K. Manstead A. S. (2004b). The interpersonal effects of emotions in negotiations: a motivated information processing approach. *J. Pers. Soc. Psychol.* 87 510–528. 15491275 10.1037/0022-3514.87.4.510

[B105] Van Kleef G. A. Van Doorn E. A. Heerdink M. W. Koning L. F. (2011). Emotion is for influence. *Eur. Rev. Soc. Psychol.* 22 114–163.

[B106] Venkiteswaran S. Sundarraj R. P. (2020). How angry are you? Anger intensity, demand and subjective value in multi-round distributive electronic negotiation. *Group Decis. Negot.* 30 143–170.

[B107] Walther J. B. (2015). “Social information processing theory (CMC),” in *The International Encyclopedia of Interpersonal Communication*, eds Berger C. R. Roloff M. E. (Hoboken, NJ: John Wiley & Sons), 1–13.

[B108] Walther J. B. D’Addario K. P. (2001). The impacts of emoticons on message interpretation in computer-mediated communication. *Soc. Sci. Comput. Rev.* 19 324–347.

[B109] Wang L. Northcraft G. B. Van Kleef G. A. (2012). Beyond negotiated outcomes: the hidden costs of anger expression in dyadic negotiation. *Organ. Behav. Hum. Decis. Process.* 119 54–63.

[B110] Warschauer M. (2013). Comparing face-to-face and electronic discussion in the second language classroom. *CALICO J.* 13 7–26.

[B111] Watson D. Clark L. A. Tellegen A. (1988). Development and validation of brief measures of positive and negative affect: the PANAS scales. *J. Pers. Soc. Psychol.* 54 1063–1070. 3397865 10.1037//0022-3514.54.6.1063

[B112] Weiss H. M. Cropanzano R. (1996). Affective events theory: a theoretical discussion of the structure, causes and consequences of affective experiences at work. *Res. Organ. Behav*. 19 1–74.

[B113] Wilson K. S. DeRue D. S. Matta F. K. Howe M. Conlon D. E. (2016). Personality similarity in negotiations: testing the dyadic effects of similarity in interpersonal traits and the use of emotional displays on negotiation outcomes. *J. Appl. Psychol.* 101 1405–1421. 27336910 10.1037/apl0000132

[B114] Yip J. A. Schweinsberg M. (2017). Infuriating impasses: angry expressions increase exiting behavior in negotiations. *Soc. Psychol. Pers. Sci.* 8 706–714.

[B115] Yun D. Jung H. Ashihara K. (2020). Dimensions of leader anger expression unveiled: how anger intensity and gender of leader and observer affect perceptions of leadership effectiveness and status conferral. *Front. Psychol.* 11:1237. 10.3389/fpsyg.2020.01237 32719630 PMC7351527

[B116] Zhou R. Hentschel J. Kumar N. (2017). “Goodbye text, hello emoji: mobile communication on WeChat in China,” in *Proceedings of the 2017 CHI Conference on Human Factors in Computing Systems*, (New York, NY: Association for Computing Machinery), 748–759.

