# Corrigendum: Anger expression in negotiation: The effects of communication channels and anger intensity

**DOI:** 10.3389/fpsyg.2022.1125275

**Published:** 2023-01-05

**Authors:** Dongwon Yun, Heajung Jung

**Affiliations:** ^1^William F. Harrah College of Hospitality, University of Nevada, Las Vegas, Las Vegas, NV, United States; ^2^School of Business, Konkuk University, Seoul, South Korea

**Keywords:** anger expression, negotiation, communication channel, anger intensity, non-verbal cues

In the published article, there was an error regarding the affiliation for author Dongwon Yun. As well as having affiliation “1 William F. Harrah College of Hospitality, University of Nevada, Las Vegas, Las Vegas, NV, United States,” they should also have affiliation, “2 School of Business, Konkuk University, Seoul, South Korea.”

The authors apologize for this error and state that this does not change the scientific conclusions of the article in any way. The original article has been updated.

